# Detection of short-term activity avalanches in human brain default mode network with ultrafast MR encephalography

**DOI:** 10.3389/fnhum.2015.00448

**Published:** 2015-08-11

**Authors:** Zalán Rajna, Janne Kananen, Anja Keskinarkaus, Tapio Seppänen, Vesa Kiviniemi

**Affiliations:** ^1^Biomedical Engineering Research Group, Department of Computer Science and Engineering, Faculty of Information Technology and Electrical Engineering, University of OuluOulu, Finland; ^2^Oulu Functional Neuroimaging Research Group, Department of Diagnostic Radiology, Medical Research Center, Oulu University HospitalOulu, Finland

**Keywords:** activity avalanche detection, human brain, default mode network, resting state, functional magnetic resonance imaging

## Abstract

Recent studies pinpoint visually cued networks of avalanches with MEG/EEG data. Co-activation pattern (CAP) analysis can be used to detect single brain volume activity profiles and hemodynamic fingerprints of neuronal avalanches as sudden high signal activity peaks in classical fMRI data. In this study, we aimed to detect dynamic patterns of brain activity spreads with the use of ultrafast MR encephalography (MREG). MREG achieves 10 Hz whole brain sampling, allowing the estimation of spatial spread of an avalanche, even with the inherent hemodynamic delay of the BOLD signal. We developed a novel computational method to separate avalanche type fast activity spreads from motion artifacts, vasomotor fluctuations, and cardio-respiratory noise in human brain default mode network (DMN). Reproducible and classical DMN sources were identified using spatial ICA prior to advanced noise removal in order to assure that ICA converges to reproducible networks. Brain activity peaks were identified from parts of the DMN, and normalized MREG data around each peak were extracted individually to show dynamic avalanche type spreads as video clips within the DMN. Individual activity spread video clips of specific parts of the DMN were then averaged over the group of subjects. The experiments show that the high BOLD values around the peaks are mostly spreading along the spatial pattern of the particular DMN segment detected with ICA. With also the spread size and lifetime resembling the expected power law distributions, this indicates that the detected peaks are parts of activity avalanches, starting from (or crossing) the DMN. Furthermore, the split, one-sided sub-networks of the DMN show different spread directions within the same DMN framework. The results open possibilities to follow up brain activity avalanches in the hope to understand more about the system wide properties of diseases related to DMN dysfunction.

## Introduction

A neuronal activity avalanche is a cascade of bursts of activity in the nervous system. It has been stated that although this phenomenon is highly robust and reproducible, its relation to physiological processes in the intact brain is currently not known (Beggs, 2007). Previous works from Beggs and Plenz (2004) described the activity avalanches at the maximum spatial scale of local field potentials (LFPs). It was shown for neuronal avalanches, that the spatial and temporal distributions can be well described by power laws, which indicates that the propagation of spontaneous activity in cortical networks are organized into a critical state in which event sizes show no characteristic scales (Beggs and Plenz, 2003).

Palva and co-workers were able to use MEG/EEG (MEEG) to pinpoint visually cued networks of avalanches (Palva et al., 2013). However, the accuracy of this method is limited spatially to the brain surface, while fMRI is a natively three dimensional technique in space. It has been shown in fMRI data, that avalanches of activity are ruled by the same dynamical and statistical properties described previously for neuronal events at smaller scales (Tagliazucchi et al., 2012). Also in fMRI data, Liu and Duyn developed a co-activation pattern (CAP) analysis in order to detect single brain volume activity profiles and hemodynamic fingerprints of neuronal avalanches as sudden activations of networks (Liu and Duyn, 2013). Yet, activity avalanche detection has not been performed on temporally ultra-fast sampled BOLD recordings like magnetic resonance encephalography (MREG).

Classical 2 s TR BOLD scanning is slow in temporal resolution causing aliasing of physiological signals into lower frequencies of interest for instance. MREG is a novel BOLD MR sequence that achieves a higher, 100 ms whole brain sampling interval. It has been proven (Jacobs et al., 2014b) that MREG data have higher statistical power for analyzing for instance epileptic networks than previously used sequences. Higher sensitivity compared to conventional sequences in recent findings considering epilepsy has also been shown (Jacobs et al., 2014a), and ultra-fast partial k-space trajectory sequences allow a 20 times faster data sampling rate (Assländer et al., 2013).

MREG therefore allows a more accurate spatio-temporal profiling of the activity spread, even with the inherent hemodynamic delay of the BOLD signal; if two connected or neighboring regions activate one after the other, the measured signal can follow the spread of neuronal activity to connected or neighboring areas (Ogawa et al., 2000; Tomatsu et al., 2008; Sung and Ogawa, 2009). There is some criticism to this due to variations in hemodynamic delays, especially in causality analytics. However, considering a local spread, the hemodynamic delay is small and allows accurate spatial estimations of avalanches (Misaki et al., 2013).

The presence of CAP's in fMRI data was initially described within the default mode network (DMN). The DMN includes precuneus/posterior cingulate cortex, medial prefrontal cortex and medial, lateral and inferior parietal cortex. The DMN splits often in high model order ICA (Abou-Elseoud et al., 2010). It is a consistent pattern of deactivation across a network of brain regions that occurs during the initiation of task-related activity (Raichle et al., 2001). As stated by Broyd et al., the DMN concept, although only first introduced into the published literature in 2001, has rapidly become a central theme in contemporary cognitive and clinical neuroscience (Broyd et al., 2009). DMN dysfunction has been found to be related to dementia, schizophrenia, epilepsy, anxiety and depression, autism and attention deficit/hyperactivity disorder (Broyd et al., 2009). The DMN splits hierarchically in high model order ICA into several sub-networks (Abou-Elseoud et al., 2010) and sliding window ICA in the DMN has marked spatial dynamics even from frame to frame (Kiviniemi et al., 2011).

Tagliazucchi et al. have shown that resting state classical 2.5 s TR BOLD activity between two inactive phases with an isolated start of activation shows avalanche type properties (Tagliazucchi et al., 2012). Liu and Duyn analyzed classical 2 s TR BOLD signal avalanche fingerprints in DMN (Liu and Duyn, 2013). The ultra-fast sampling rate of MREG offers the possibility to detect the spatial and temporal spread of these and even faster avalanche type activity spreads. However, since the MREG signal is very sensitive to physiological noise, it is required to develop advanced techniques to remove noise components such as motion artifacts, vasomotor fluctuations, and cardio-respiratory noise in order to detect accurately the activity avalanches. We present a computational pipeline, comprising several steps to filter the signal and separate the avalanche type activity spreads in the DMN from physiological noise.

## Methods

**Figure 1:**
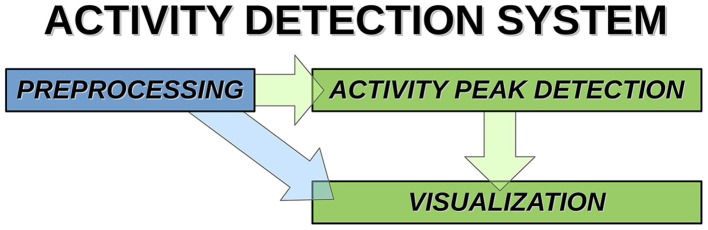
**System components and data paths of activity detection**.

Overview of the activity avalanche detection system is shown in Figure 1.

### Measurement data

The MR system is a Siemens 3T SKYRA with a 32-channel head coil. MREG sequence obtained from Freiburg University via collaboration with Jürgen Hennig group (Zahneisen et al., 2012; Lee et al., 2013) was utilized. MREG is a three-dimensional (3D) spiral, single-shot sequence that undersamples 3D k-space trajectory for faster imaging (Assländer et al., 2013). It samples the brain at 10 Hz frequency (TR = 100 ms, TE = 1.4 ms, and flip angle = 25°) and offers thus about 20–25 times faster scanning than conventional fMRI. Three-dimensional MPRAGE (TR = 1900 ms, TE = 2.49 ms, flip angle = 9°, FOV = 240, and slice thickness = 0.9) images were used to register the MREG data into 4 mm MNI space.

Data was collected from 10 min long resting state MREG measurements from 11 healthy control subjects (3 women, 27.2 ± 7.5 years old). The study protocol was approved by the ethics committee of the Northern Ostrobothnia Hospital District. Written informed consent was obtained from each subject individually prior to scanning, in accordance with the Helsinki declaration. During the 10-min MREG resting-state study, subjects were instructed to lie quietly in the scanner with their eyes open fixating at a cross on the screen and thinking nothing particular. As criteria for the selection of appropriate recordings for further study, both completeness of data as well as performance against physiological noise removal by DRIFTER (Särkkä et al., 2012) software were used.

### Preprocessing pipeline

**Figure 2:**
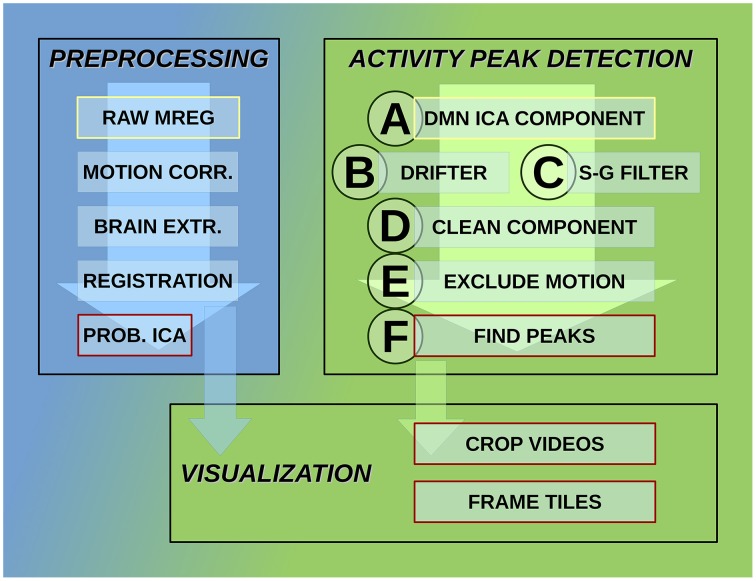
**Steps of the activity detection system**. Preprocessing steps for raw MREG data: motion correction, brain extraction, image registration, and probabilistic independent component analysis across subjects resulting in components part of the DMN as well. Activity peak detection on each DMN ICA component: reduce cardio-respiratory physiological noise and calculate slow vasomotor fluctuations, clean ICA component from physiological noise, exclude time points and neighborhood affected by head motion, peak detection on clean and motion-free signal. Visualization using data after registration preprocessing step: crop MREG data around activity peaks and extract slices as videos and frame tiles in the spatial region of interest.

MREG data are being preprocessed with FSL pipeline in the same way as described by Korhonen et al. (2014). One hundred and eighty time points are removed from the beginning for minimizing T1-relaxation effects. Head motion was corrected with FSL 5.01 MCFLIRT software (Jenkinson et al., 2002). The translational and rotational movement of the subjects was used in this study to exclude motion artifacts while detecting activity avalanche peaks. The overview of the detection system steps is shown in Figure 2.

Brain extraction was carried out after MCFLIRT with optimization of the deforming smooth surface model, as implemented in FSL 5.01 BET software (Smith, 2002) using threshold parameters *f* = 0.3 and *g* = 0; and for 3D MPRAGE volumes, using parameters *f* = 0.25 and *g* = 0.22 with neck and bias field correction option. Spatial smoothing was done with fslmaths 5-mm FWHM Gaussian kernel. Three-dimensional MPRAGE images were used to register the MREG data into MNI space in 4-mm resolution using MELODIC (Beckmann and Smith, 2004) for group independent component analysis (ICA).

To separate resting state networks (RSN), such as DMN, probabilistic ICA (PICA) was calculated for MREG data as implemented in MELODIC. Group ICA was used to separate noise sources from RSN sources with previous criteria (Kiviniemi et al., 2003, 2009), model order was chosen to be 70. The activity avalanche detection was performed on the selected ICA components with analysis in time dimension.

### Activity peak detection

**Figure 3:**
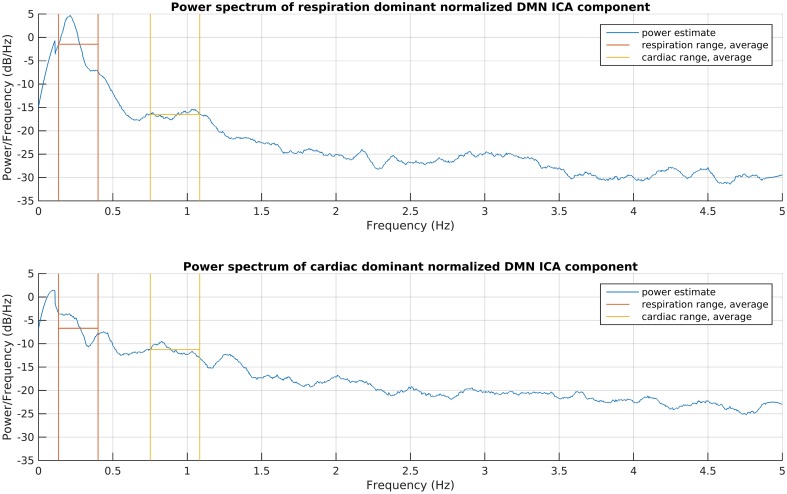
**Power spectrum estimations of the introduced respiration and cardiac noise dominant DMN ICA components**. The signals were taken for the whole recording and normalized to zero mean and one variance before spectrum estimations, to become comparable. The frequency ranges given for DRIFTER for physiological noise filtering are shown together with the power average in those ranges.

**Figure 4:**
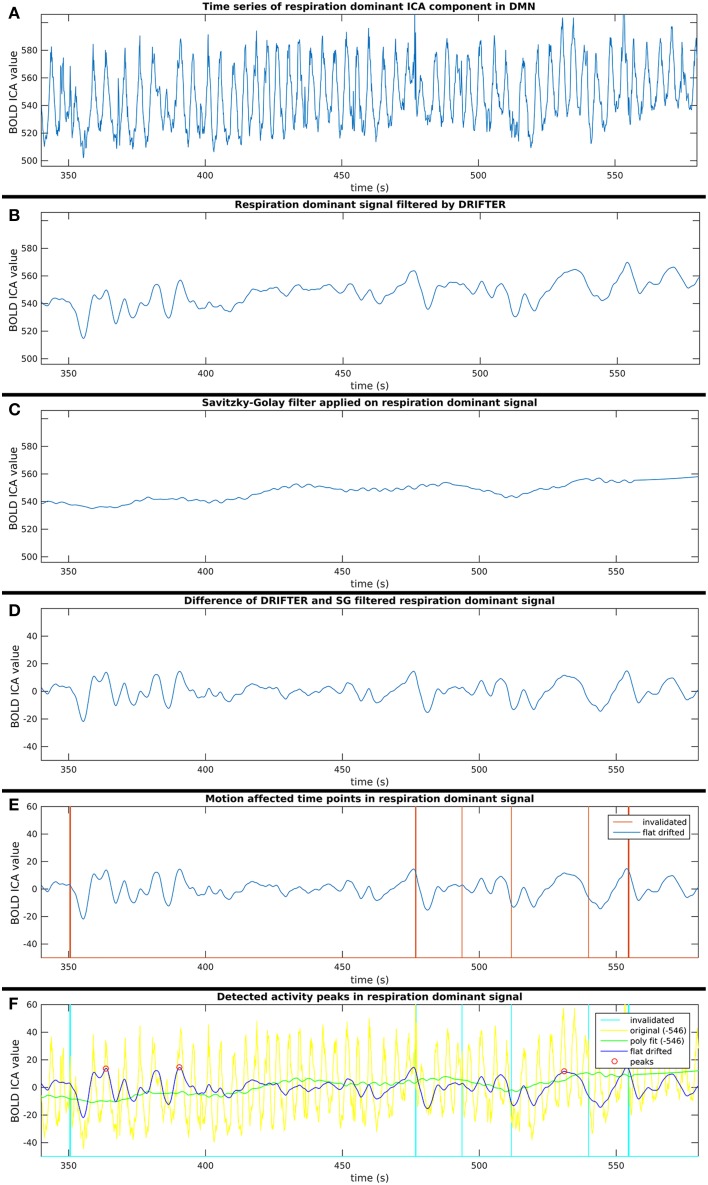
**Six step process (A–F) of activity peak detection: example on a 4 min time window of an ICA component dominated by respiration physiological noise, also shown in Figure 3**.

**Figure 5:**
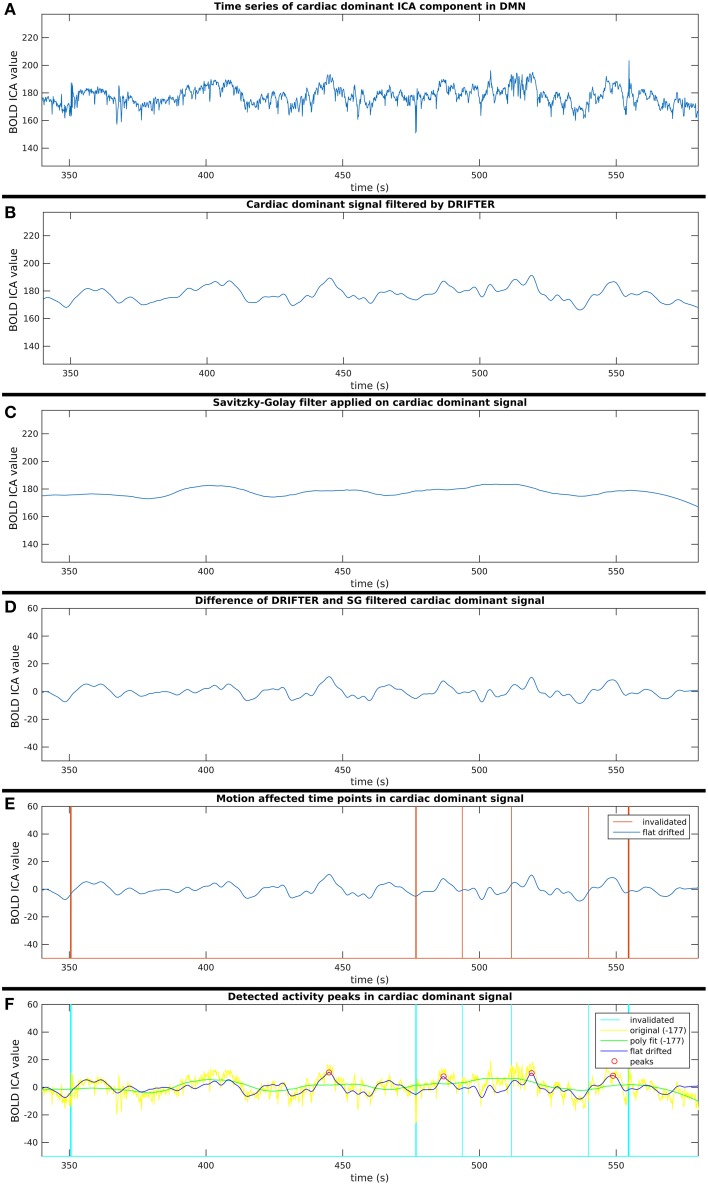
**Six step process (A–F) of activity peak detection: example on a 4 min time window of an ICA component dominated by cardiac physiological noise, also shown in Figure 3**.

This process consists of six steps (A-F), as shown in Figure 2, and they are introduced with two example components. The power spectra of two example ICA components, selected from the same subject, are shown in Figure 3. The six steps (A-F) are demonstrated separately in Figures 4, 5 for respiration and cardiac physiological dominant signals, respectively.

#### Step A: selection of ICA components in DMN

The ICA components, obtained with preprocessing, as explained in Section Preprocessing Pipeline, were analyzed by an ICA resting state neuroradiologist (Kiviniemi et al., 2003, 2009), and five components dominated by anatomical areas of posterior cingulate cortex and (ventro)medial prefrontal cortex of the DMN were selected. One component representing medial prefrontal cortex (DMN_mpf_), one representing ventromedial prefrontal cortex (DMN_vmpf_) and three split ICA components representing posterior cingulate cortex (DMN_pcc_) were selected (Abou-Elseoud et al., 2011). To assure convergence of ICA method and spatial accuracy of the DMN components, physiological noise removal, and motion detection were performed after this step. Further data processing (steps B-F) on the selected ICA components in the DMN was done with custom-made software implemented in Matlab (Release 2014b. The MathWorks Inc. Natick, Massachusetts, United States).

#### Step B: respiration and cardiac physiological noise removal with DRIFTER

First, physiological noise removal was done for each original time series of the selected group ICA components as separate runs with DRIFTER, which is a Bayesian method for retrospective elimination of physiological noise from fMRI data. In this case, the main advantage of using DRIFTER compared to other methods like RETROICOR (Glover et al., 2000) was the possibility of physiological noise removal without reference signals, only defining estimated frequency ranges. The algorithm first estimates the frequency trajectories of the physiological signals with the interacting multiple models (IMM) filter. The frequency trajectories in this case were estimated from the fMRI data since temporal resolution was high enough (*f*_*s*_ = 10 Hz). Frequency ranges for trajectory estimation as seen on Figure 3 were 8–24 bpm (0.13–0.4 Hz) with 0.2 bpm (0.0033 Hz) steps and 45–65 bpm (0.75–1.08 Hz) with 0.4 bpm (0.0067 Hz) steps for respiratory and cardiac noise, respectively. The estimated frequency trajectories are then used in a state space model in combination of a Kalman filter (KF) and Rauch–Tung–Striebel (RTS) smoother, which separated the signal into an activation related cleaned signal, physiological noise, and white measurement noise components (Särkkä et al., 2012).

DRIFTER physiological noise removal method did not succeed for all components. There were more 10 min long MREG recordings for which DRIFTER succeeded in all five components (see Section Step A: Selection of ICA Components in DMN), therefore, the broad spatial distribution of an ICA component could not be the reason behind it. The phase shifts of the dominant frequencies to track made the IMM filter of DRIFTER succeed at 65.5% of the data. Successful outputs per subjects were varying between one and five. To evaluate the quality of the frequency trajectory estimation, own scores (referred later as “Dscores”) were introduced which reflect the standard deviation of the frequency values of the trajectory. This method was derived from the experimental observation that noise removing performance was poor when the trajectory tracking approached the minimum or maximum of the predefined frequency range quickly, resulting in low variance of frequency values of the trajectory estimation at the same time. Experimental limit for scores with good noise removing performance was one, which was exceeded by 97.3% of the DRIFTER outputs.

#### Step C: removal of vasomotor fluctuations with savitzky–golay filter

Meanwhile, slow vasomotor fluctuations were estimated also from the original time series of the selected group ICA components with the Savitzky–Golay polynomial filter (polynomial order 2, window size 513) for which over 80% of the power of these estimated signals were under 10^−2^ Hz. This filter minimizes least-squares error on the given time window with the polynomial fit, and is widely used on physiological data (Hargittai, 2005).

#### Step D: cleaning components from noise identified in steps B and C

Short-term avalanche type activity spreads appear on top of the “base activity,” thus, the activity peak detection needed to be independent from the vasomotor fluctuations. Therefore, the polynomial smoothed signal was subtracted from the signal resulted by the cardio-respiratory physiological noise removal by DRIFTER. With this step, we are both removing vasomotor fluctuations and physiological noise while normalizing the signal to a close to zero mean, which enables to compare group ICA components appearing in different spatial (anatomical) areas.

#### Step E: Exclusion of potentially motion affected time points

**Figure 6:**
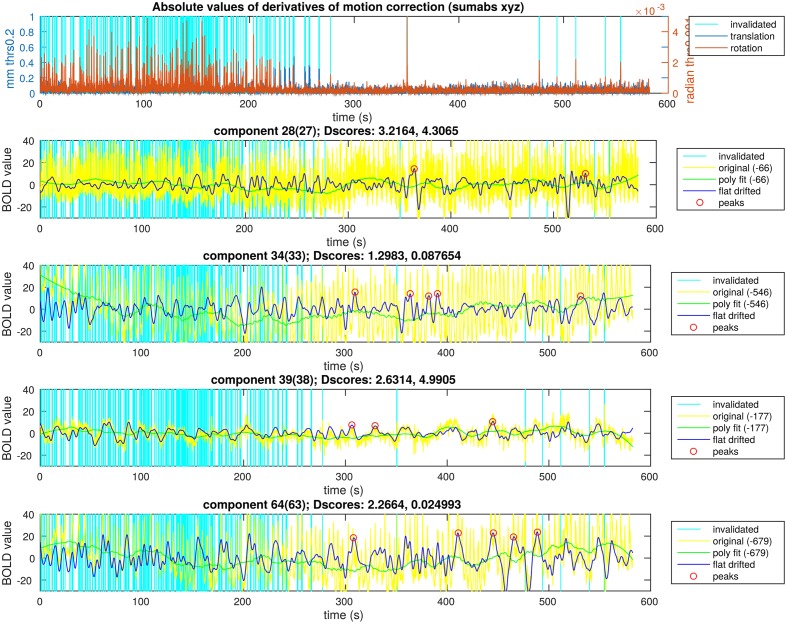
**Peak detection result overview for a single subject**. The motion data with the time points selected to exclude are in the first plot. In the following four plots there are ICA components of a single subject and the steps of the activity peak detection as an overview in each plot, also containing the detected activity peaks.

For each time signal, the motion correction data of the MREG measurement was used to exclude time points around motion artifacts. Velocity information was calculated by differentiating translational and rotational correction data along time dimension. Separately, the absolute values of translational and rotational velocity data were summed in all three MNI spatial dimensions, resulting in a translational and rotational speed representation. An example of results is illustrated in Figures 4–6. Time points and their neighborhood, where the sum of translational speed exceeded 0.2 mm per sample, or the rotational speed exceeded 10^−3^ radians per sample were excluded. In order to confirm that the results are clean from motion artifacts, the excluded neighborhood was the same as the range of later cropped MREG data around found peaks. This is 5 s preceding and 10 s following the detected activity peak. This particularly strict method results in the exclusion of 77% of the detected peaks with the remaining signal representing motion-free data.

#### Step F: finding activity peaks in clean time series

Peak detection was performed on the clean signal. Peaks with higher value than the 150% of the signal standard deviation were initially marked. The minimum distance between found peaks was 50 samples (5 s), and not the first, but always the highest peak within the minimum distance (search window) was marked.

### Size and lifetime of an activity peak

Using MREG data, which is critically sampled for cardiac and respiratory physiological noise, introduces new possibilities, but also limitations for previously used methods. One of these is, that it measures physiological signals so accurately, that methods described earlier to define size and length of an activity spread around a peak (Tagliazucchi et al., 2012) would only measure respiration fluctuations at many anatomical points in the brain, including parts of the DMN. As stated earlier, it is currently not possible to clean the four dimensional BOLD data directly from physiological noise without unacceptable data loss, which is also the reason for searching activity peaks in ICA time series. Thus, we needed to develop own methods to estimate size and lifetime of activity spreads belonging to found peaks in the clean ICA components.

The size of an activity spread was estimated by the sum of the absolute values of normalized BOLD ICA values during the spreading lifetime in the single clean ICA time series where the activity peak was detected. The lifetime of an activity spread was defined as the widest continuous time range around the detected peak where no value falls under the peak detection threshold (see Section Step F: Finding Activity Peaks in Clean Time Series). This estimation is clearly limited (due to the limitations described in the previous paragraph), because activity spreads are not local to the spatial distribution of an ICA component, but the power reaching the area still enables us to make a rough estimation of size and lifetime.

### Visualization

The steps described in Section Activity Peak Detection result in peak detection, and furthermore reflect avalanche type activity spreads (see Section Single Activity Avalanches). The retrieved information was used for processing registered MREG data (registration step in preprocessing, Figure 2) to visualize the obtained results. First of all, the BOLD values were normalized for the whole time length to zero mean and one variance. This was done for effective visualization and valid comparison. The aim of the visualization was to inspect spatial spread and distribution of the avalanches detected from the clean signal, which on top of a standard anatomical image allows observation of MREG data.

For all detected peaks, the MREG data was cropped in the following way: 50 samples preceded and 100 samples followed the sample of the activity peak. This leads to 151 time samples (15.1 s) long four dimensional MREG data for all detected peaks. Three orthogonal slices were observed according to MNI space for which the spatial intersection point was the highest value in the spatial distribution of the actual ICA component within the DMN. The normalized and color coded MREG data was overlaid on a standard anatomy image. Color coding was the following with the three traditional color channels: red at maximum in case the voxel value is positive, blue at maximum in case the voxel value is negative, and green channel at the normalized absolute value of the actual voxel. This results in red to yellow and blue to cyan color scales for positive and negative values, respectively.

## Results

After finding the activity peaks, it was verified that the found peaks really refer to an activity flash in the selected DMN areas in an average case, and the peaks are not just a side effect of neighboring activities from which only parts can be seen through the spatial masks of the ICA components. For this, the peak videos were averaged, as explained in Section Group Mean Activity.

In Figure 6, we illustrate the result of an activity peak detection for a single subject. One of the important goals was the complete rejection of the motion affected time points. In this example, the first 4 min of motion estimation showed a lot of head motion exceeding our very low threshold. We can see time points with similarly high activity peaks in the starting region as the points marked in the second half of the recording, but the peaks around possible head motion were not marked. In the motion free time range, we can observe time points unmarked, with similarly high activity peaks as the marked ones. This is to prevent overlapping during the visualization, thus, only the highest peak within a certain search window was marked. This is not the case across different components, because the overlapping is not a disturbing issue while visualizing the activity avalanches in different planes for different components. With keeping these two exceptions in mind, we can observe in Figure 6 that clearly the highest activity peaks were marked for visualization.

### Single activity avalanches

**Figure 7:**
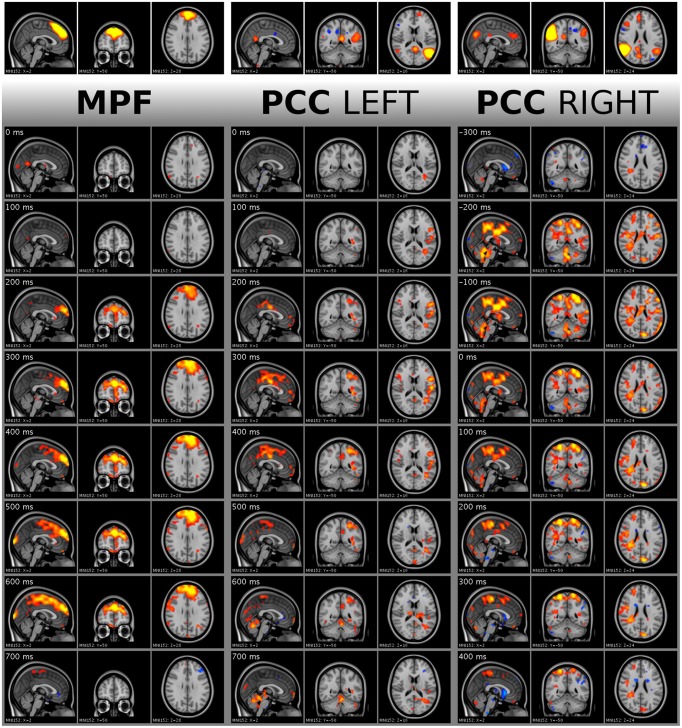
**Visualization of single activity avalanches in DMN_mpf_ and DMN_pcc_ by frame tiles with vertical time flow (from top to bottom) and 100 ms frame distances**. The top frames are the analyzed ICA components cut and saturated at z-score thresholds 3 and 7, respectively. The vertically following frames are normalized BOLD data, cut and saturated at absolute threshold value 1.7 and 3.5, respectively. Values are color coded from low to high as red to yellow and blue to cyan, for positive and negative values, respectively. Activity data was smoothed with a Gaussian kernel (mean 0, standard deviation 1.2 pixels), and the voxel values outside the brain set to zero. Activity avalanche in DMN_mpf_ and in DMN_pcc left_ starts at frame three, in DMN_pcc right_ starts at frame two. All three shown activity avalanches are five frames long. The BOLD values are spatially registered and thus overlaid on a standard anatomy image.

For every subject, five DMN ICA components were processed. Even with the strict exclusion of the possibly motion affected time points, 130 single activity peaks were found. However, a sample point of an activity peak is not perfectly punctual because, as a result of smoothing in DRIFTER, the peak might be shifted with several samples. To obtain an estimation of this error, the starting points of activity spreads were manually observed. As in the example of Figure 7, the starting point of an activity spread can be clearly seen in the concerned anatomical area with global thresholds over all subjects and components. By manual inspection of such extracted frame tiles, the actual difference of the activity spread starting point from frame zero was determined. Thus, the standard deviation around frame zero of the starting points resulted in 4.20 frames (0.42 s).

Similarly to the starting point, the length of the activity avalanche can be also manually observed in the four dimensional data. This is different from the lifetime estimation, which is computed, and not a manual observation. The standard deviation of the activity avalanche lengths was 2.33 frames (0.233 s) around the mean value of 3.75 frames (0.375 s). This is a length of activity which can only be observed with ultrafast sampling MR sequences like MREG. The short length also excludes the thought that the effect of the cardiac or respiratory noise would be seen directly, since such a rapid change is in a higher frequency range than the heart rate or respiration of a resting individual.

The activity spreads were analyzed by a resting state specialist, and quick, avalanche type activity spreading was observed. In Figure 7, the activity spread in DMN_mpf_ is a typical detected phenomenon. The spreads in DMN_pcc left_ and DMN_pcc right_ demonstrate the left and right splitting of DMN ICA components and their different activity spread directions.

**Figure 8:**
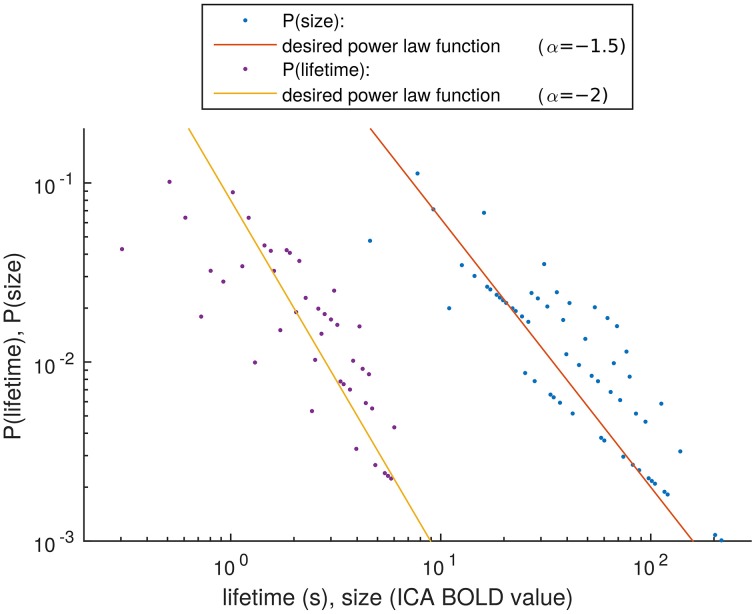
**Distribution estimation of size and lifetime of activity spreads**. The desired power law functions are marked with lines.

The distribution estimations of the size and lifetime of an activity peak are shown in Figure 8. These were calculated as described in Section Size and Lifetime of an Activity Peak. It has been proven in previous works, that activity spreads extracted around BOLD peaks have avalanche type properties (Tagliazucchi et al., 2012; Liu and Duyn, 2013). With this small number of detected activity spreads it is not possible to verify completely the avalanche properties described previously (Beggs and Plenz, 2003; Tagliazucchi et al., 2012). However, we apply a very similar definition of activity spreads as is used for avalanches, i.e., selecting high data values preceded and followed by still data. With this definition, the resulting trends for the detected activity spreads are as expected from theory for avalanches (marked by lines in Figure 8), supporting our choice to call the activity around the detected peaks as avalanche type activity spreads. With the small amount of data, the roughness of the size, and lifetime estimates could be responsible for the many outliers, but there is no better method currently available for critically sampled fMRI data, i.e., where the cardiac and respiration signals do not alias into lower frequencies.

### Group mean activity

**Figure 9:**
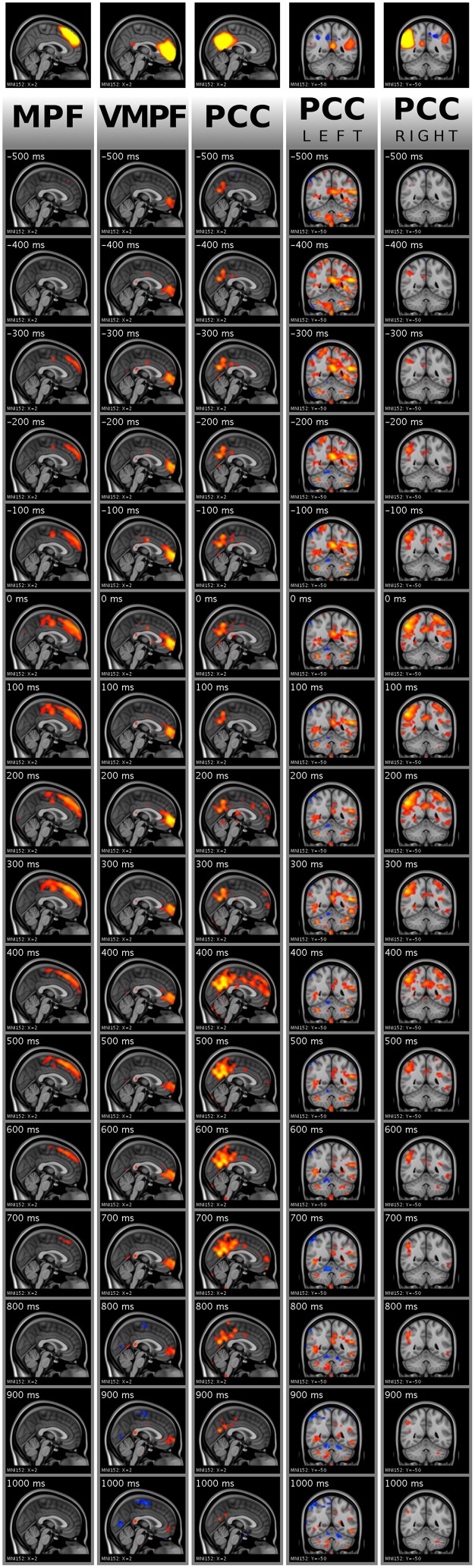
**Visualization of activity avalanche group means for all selected ICA components in DMN_(v)mpf_ and DMN_pcc_ by frame tiles in single planes with vertical time flow (from top to bottom) and 100 ms frame distances**. The top frames are the analyzed ICA components cut and saturated at z-score thresholds 3 and 7, respectively. The vertically following frames are normalized BOLD data, cut and saturated at absolute threshold values, from left to right: 1 and 2; 0.8 and 1.5; 0.8 and 1.5; 0.4 and 0.8; 0.8 and 1.5, respectively. Values are color coded from low to high as red to yellow and blue to cyan, for positive and negative values, respectively. Activity data was smoothed with a Gaussian kernel (mean 0, standard deviation 1.2 pixels), and the voxel values outside the brain set to zero. The BOLD values are spatially registered and thus overlaid on a standard anatomy image.

From the single peaks, an average was created for all ICA component separately, i.e., cropped data around all found activity peaks in a single component of a single subject was averaged among peaks into a single 151 frames long (15.1 s) data set. As a second step, with equal weight for each subject, a group mean was calculated along subjects resulting in five 151 frame long data sets representing the originally selected five DMN ICA components. In Figure 9, the group mean frames for all five ICA components around the peak detection point are shown in a selected plane.

As explained in Section Single Activity Avalanches, the signal smoothing during physiological noise removal may result in few frames shift of the activity peak. Therefore, the mean frames provide dominant spatial information of the avalanche type activity spreads instead of holding accurate timing information. Importantly, we observed that the avalanches belonging to DMN_pcc_ components split to the left and the right side are dominant on different sides of the brain, according to the original ICA component. We provide the whole 151 frame data for the DMN_(v)mpf_ and DMN_pcc_ components presented in both Figures 7, 9 in video format as Supplementary Material. Note, that visualization data is not clean from physiological noise.

For the group mean data, the spatial focus of high BOLD activity remains in the region of interest anatomically. Thus, the activity spreads, including activity avalanches, started from or at least crossed this anatomical area.

## Discussion and future prospects

Activity avalanches and their momentous fingerprint CAP's in fMRI have recently been detected with classical BOLD scans of 2(-2.5) s TR (Tagliazucchi et al., 2012; Liu and Duyn, 2013; Palva et al., 2013). Our results show that by combining ultra-fast MREG imaging with advanced signal processing, one can detect spatial spread of avalanche type brain activity with 100 ms time-frame accuracy. A key issue is the proper detection of RSN signal sources prior to excess MREG signal filtering, which seem to diminish the skewness and kurtosis of RSN sources for ICA to separate them from the data. After that, motion and other noise sources need to be excluded for the detection of suitable peaks for avalanche type activity spreads in the brain. Even though the BOLD signal has inherent hemodynamic delay, one can still detect successive spread of neuronal event within sufficiently similar regions (Ogawa et al., 2000; Tomatsu et al., 2008; Sung and Ogawa, 2009).

Importantly, the activity spread needs to be distinguished from cardiac and respiratory pulsations. Our ultra-fast MREG system enables critical sampling of the cardio-respiratory noise that can be extracted from the signal with some prerequisites. Also our preliminary QPP analyses suggest markedly differential spatio-temporal spread of cardio-respiratory pulsations in the human brain and thus also exclude their participation as a source in signal change.

ICA has become one of the main tools in separating brain activity sources, whether activated, spontaneous or inherent in origin (McKeown and Sejnowski, 1998; Calhoun et al., 2001; Kiviniemi et al., 2003; Greicius et al., 2004; Beckmann et al., 2005). One puzzling question has been the splitting of the detected brain networks, including the DMN, into several sub-networks with increasing model orders (Ma and Wang, 2008; Abou-Elseoud et al., 2010, 2011). Splitting has been suggested to be a sign of over-fitting the ICA model, but our results suggest, at least in model order 70, an activity avalanche based origin for the splitting. Figures 7, 9 (along with Supplementary Videos) illustrate that within these networks, the avalanches type activity spreads are indeed different in the split sub-networks, emphasizing the avalanche activity on the side of the detected sub-network. Therefore, the spatial ICA splitting in higher model orders seems to be a sign of neuronal activity spread difference of separate events. In lower model orders, these events become grouped together as a lower hierarchical system due to summed explanation of variance.

One of the main difficulties in obtaining clear results is that the physiological noise is hardly separable from higher frequency neuronal activity. Our novel idea was to use the DRIFTER for the peak detection only on single time series, and not already on the reconstructed MREG signal (see preprocessing step), after which ICA analysis leads to inaccurate components. As a result, we cleaned the already spatially accurate group ICA output signals from vasomotor fluctuations and cardio-respiratory physiological noise. This indeed involved many steps, but also showed the richness of MREG data and the use of its high time resolution. By showing, that the group mean of activity peaks and their neighborhood keep their focus on the anatomical area of the ICA component in the DMN, and individually high activities tend to happen in a fraction of a second, we consider found time peaks belonging to avalanche type activity spreads, which when cleaned from other physiological effects, must be activity avalanches.

Current tools defining spatial accuracy are not perfect. If the registration of the MREG data on the brain anatomy could be done more precisely, and the spatially sparse parts of a single ICA component could be segmented, a higher overall and DRIFTER performance would be achieved. Here, we used DRIFTER for removing physiological noise, however, experiments showed the limitations of its performance in some cases. Accordingly, also other methods instead of DRIFTER could be taken into account or compared, like temporal ICA, novel MREG sequences, or improvements on DRIFTER by using the actual measured cardiac and respiration data. A clear limitation is the number of subjects included in the study and thus the amount of data analyzed.

It is a future goal to compare the findings with other data modalities. We have collected simultaneous multimodal data, where we have scalp DC-EEG, near-infrared spectroscopy (NIRS), noninvasive blood pressure (NIBP) and full anesthesia monitoring during MREG scanning for subjects in this study, using our HEPTA-scan concept (Korhonen et al., 2014). Our aim is to model EEG source avalanches and analyze how their spread might be connected with MREG avalanches. Other modalities, like NIRS and NIBP data, could be also used to model local oxy/deoxy concentration changes and vasomotor waves around the detected points. Currently, all subjects were healthy volunteers. In the future, we plan to include and compare epileptic subjects, either as a group or individually. Considering the higher statistical power of MREG data for analyzing epileptic networks (Jacobs et al., 2014b), there is a potential in research toward using our method also for subjects with diseases.

Furthermore, a method to quantify dynamics of three dimensional spread patters would also have a lot of benefits, and would shorten the time spent on visual inspections and interpretation of the results, among improving current rough anatomical alignments. On long term this is the superior feature of MRI data in analyzing activity avalanches since the recording is performed natively in three spatial dimensions, however, this involves advanced signal processing techniques accordingly. Future work combining multi-modal EEG, NIRS, and NIBP will further increase accuracy of separating signal sources from the continuously active brain.

## Conclusion

This study has shown, that it is possible to find avalanche type activity spreads in the DMN which promise the opportunity for further analyzing activity avalanches in these regions with a natively three dimensional imaging technique in space. With the time resolution of ultra-fast MREG, activity avalanche detection has not been performed before, while comparing and following up individual activity spreads, based on this peak detection method, is in a reachable sight now. Furthermore, considering the analyzed brain areas, it is a step toward developing detection of features with diagnostic value in DMN dysfunction related diseases, which include dementia, schizophrenia, anxiety and depression, autism and attention deficit/hyperactivity disorder.

### Conflict of interest statement

The authors declare that the research was conducted in the absence of any commercial or financial relationships that could be construed as a potential conflict of interest.
