# Supplementary material for: Detection of short-term activity avalanches in human brain default mode network with ultrafast MR encephalography
Source: Front Hum Neurosci. 2015 Aug 11;9:448. doi: 10.3389/fnhum.2015.00448 (PMC4531800; doi:10.3389/fnhum.2015.00448)
Supplement: Supplementary file 9 [file DataSheet1.DOCX]

***Supplementary Material***

**Detection of activity avalanches in human brain with ultrafast MREG**

**Zalán Rajna^1*^, Janne Kananen^2^, Anja Keskinarkaus^1^, Tapio Seppänen^1^ and Vesa Kiviniemi^2^**

^1^Biomedical Engineering Research Group, Department of Computer Science and Engineering, ITEE, University of Oulu, Oulu, Finland

^2^Oulu Functional Neuroimaging Research Group, Department of Diagnostic Radiology, MRC, Oulu University Hospital, Oulu, Finland

*** Correspondence:** Zalán Rajna, Department of Computer Science and Engineering, P.O.Box 4500, 90014 University of Oulu, Finland.

zalan.rajna@ee.oulu.fi

1. **Supplementary Data**

**Supplementary Video 1. Single activity avalanche in DMN_mpf_ with 100 ms frame distances.** Overlaid on a standard anatomy image, shown is normalized BOLD data cut and saturated at absolute threshold value 1.7 and 3.5, respectively. Values are color coded for low to high as red to yellow and blue to cyan, for positive and negative values, respectively. Activity data was smoothed with a Gaussian kernel (mean 0, standard deviation 1.2 pixels), and the voxel values outside the brain set to zero. The detected time point of the activity peak is labeled with “0 ms”. This is the same data as shown in Figure 7.

**Supplementary Video 2. Single activity avalanche in DMN_pcc_ left with 100 ms frame distances.** Overlaid on a standard anatomy image, shown is normalized BOLD data cut and saturated at absolute threshold value 1.7 and 3.5, respectively. Values are color coded for low to high as red to yellow and blue to cyan, for positive and negative values, respectively. Activity data was smoothed with a Gaussian kernel (mean 0, standard deviation 1.2 pixels), and the voxel values outside the brain set to zero. The detected time point of the activity peak is labeled with “0 ms”. This is the same data as shown in Figure 7.

**Supplementary Video 3. Single activity avalanche in DMN_pcc_ right with 100 ms frame distances.** Overlaid on a standard anatomy image, shown is normalized BOLD data cut and saturated at absolute threshold value 1.7 and 3.5, respectively. Values are color coded for low to high as red to yellow and blue to cyan, for positive and negative values, respectively. Activity data was smoothed with a Gaussian kernel (mean 0, standard deviation 1.2 pixels), and the voxel values outside the brain set to zero. The detected time point of the activity peak is labeled with “0 ms”. This is the same data as shown in Figure 7.

**Supplementary Video 4. Single activity avalanche in DMN_mpf_ with 100 ms frame distances.** Overlaid on a standard anatomy image, shown is normalized BOLD data cut and saturated at absolute threshold value 1.0 and 2.0, respectively. Values are color coded for low to high as red to yellow and blue to cyan, for positive and negative values, respectively. Activity data was smoothed with a Gaussian kernel (mean 0, standard deviation 1.2 pixels), and the voxel values outside the brain set to zero. The detected time point of the activity peak is labeled with “0 ms”. This is the same data as shown in Figure 8.

**Supplementary Video 5. Single activity avalanche in DMN_vmpf_ with 100 ms frame distances.** Overlaid on a standard anatomy image, shown is normalized BOLD data cut and saturated at absolute threshold value 0.8 and 1.5, respectively. Values are color coded for low to high as red to yellow and blue to cyan, for positive and negative values, respectively. Activity data was smoothed with a Gaussian kernel (mean 0, standard deviation 1.2 pixels), and the voxel values outside the brain set to zero. The detected time point of the activity peak is labeled with “0 ms”. This is the same data as shown in Figure 8.

**Supplementary Video 6. Single activity avalanche in DMN_pcc_ with 100 ms frame distances.** Overlaid on a standard anatomy image, shown is normalized BOLD data cut and saturated at absolute threshold value 0.8 and 1.5, respectively. Values are color coded for low to high as red to yellow and blue to cyan, for positive and negative values, respectively. Activity data was smoothed with a Gaussian kernel (mean 0, standard deviation 1.2 pixels), and the voxel values outside the brain set to zero. The detected time point of the activity peak is labeled with “0 ms”. This is the same data as shown in Figure 8.

**Supplementary Video 7. Single activity avalanche in DMN_pcc left_ with 100 ms frame distances.** Overlaid on a standard anatomy image, shown is normalized BOLD data cut and saturated at absolute threshold value 0.4 and 0.8, respectively. Values are color coded for low to high as red to yellow and blue to cyan, for positive and negative values, respectively. Activity data was smoothed with a Gaussian kernel (mean 0, standard deviation 1.2 pixels), and the voxel values outside the brain set to zero. The detected time point of the activity peak is labeled with “0 ms”. This is the same data as shown in Figure 8.

**Supplementary Video 8. Single activity avalanche in DMN_pcc right_ with 100 ms frame distances.** Overlaid on a standard anatomy image, shown is normalized BOLD data cut and saturated at absolute threshold value 0.8 and 1.5, respectively. Values are color coded for low to high as red to yellow and blue to cyan, for positive and negative values, respectively. Activity data was smoothed with a Gaussian kernel (mean 0, standard deviation 1.2 pixels), and the voxel values outside the brain set to zero. The detected time point of the activity peak is labeled with “0 ms”. This is the same data as shown in Figure 8.
